# Bouveret's Syndrome: 64-Slice CT Diagnosis and Surgical Management—A Case Report

**DOI:** 10.1155/2012/701216

**Published:** 2012-11-21

**Authors:** Dinesh Sharma, Rajan Sood, Ashwani Tomar, Anupam Jhobta, Shruti Thakur, R. G. Sood

**Affiliations:** ^1^Department of Radio Diagnosis, Indira Gandhi Medical College, Shimla 171001, India; ^2^Department of Surgery, Indira Gandhi Medical College, Shimla 171001, India

## Abstract

Gastric outlet obstruction caused by duodenal impaction of a large gallstone migrated through a cholecystoduodenal fistula has been referred to as Bouveret's syndrome. We present a case of gallstone-induced duodenal obstruction in an elderly female patient, diagnosed on a 64-slice MDCT scanner. One-stage surgery, that is, stone removal and cholecystectomy, was performed resulting in relief of obstruction and complete cure. Clinical features, multidetector computed tomography (MDCT) findings, and surgical management are discussed.

## 1. Introduction

The first case of obstruction of duodenum by a gallstone was reported by Beaussier in 1770, but the syndrome came to be recognized only after Leon Bouveret published his two cases in 1896 [1]. Gallstones are completely asymptomatic in 60% to 80% of patients [2]. When they become symptomatic, biliary colic is the most frequently encountered manifestation. Patients with mild symptoms have a higher risk of developing gallstone-related complications such as acute cholecystitis, choledocholithiasis with or without cholangitis, gallstone pancreatitis or gallstone ileus [3]. One of the rarest complications of gallstone disease is biliodigestive fistula, with a frequency of less than 1% [4]. The fistula can be cholecystoduodenal (60%), cholecystocolic (17%), cholecystogastric (5%), or choledochoduodenal (5%) [5]. Gallstones can migrate into the terminal ileum through a cholecysto-duodenal fistula and cause an intestinal obstruction at this level, but they may also anchor at the duodenum and produce the symptoms of gastric outlet obstruction [6].

 Since this syndrome is usually observed in older patients with poor medical status, a nonsurgical approach such as endoscopic stone removal has been used as the first-line treatment. The main purpose of surgical intervention should be to remove the obstructing stone by enterotomyotomy without cholecystectomy and cholecystoduodenal fistula breakdown to minimize the risks of surgery [7]. However, when necessary as the case presented here by, gallbladder excision can be performed successfully.

## 2. Case Report

A 60-year-old female presented with a two-week history of occasional vague abdominal pain that, over a period of four days, had become more severe and localized to the right upper quadrant of the abdomen. This was associated with vomiting, which relieved pain. There was history of dyspepsia for the past 4 years. On examination, the patient was mildly dehydrated without any icterus. The abdomen was distended and tender without evidence of hernia. Bowel sounds were present. Ryle's tube aspirate was bilious and around 1 litre/24 hours. Plain radiographs of abdomen and chest were normal. Potassium and sodium levels were decreased (3.2 and 124 milli equivalent/litre). Amylase was slightly raised to the level of 125.5 IU/L. Urea and creatinine levels were 84 and 1.7 milligram/dL. Her haemoglobin level was 11.2 g/dL. The white cell counted 10 × 10^9^/L. Liver function tests were normal.

**Figure 1:**
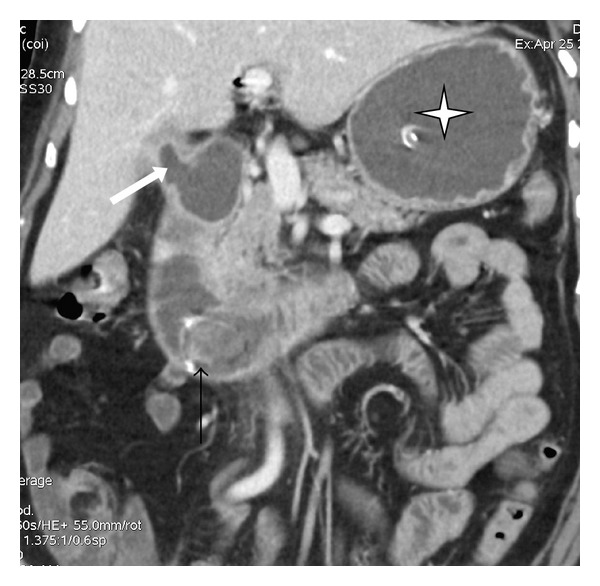
Contrast enhanced coronal reformatted MDCT image shows the cholecystoduodenal fistula (thick-white arrow) and calculus (thin-black arrow) in dilated third part of duodenum. Dilated fluid filled stomach is also seen (star).

**Figure 2:**
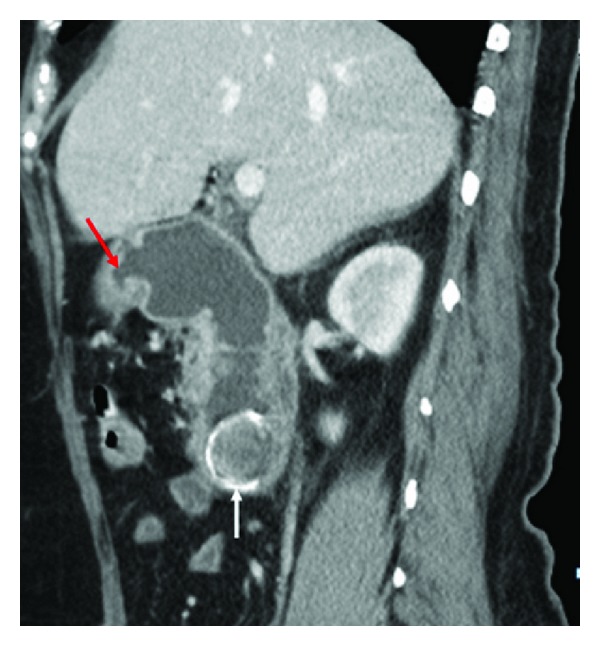
Sagittal reformatted MDCT image shows the fistula (red arrow) lying anteriorly and the calculus (white arrow) with a hyperdense rim.

Ultrasound examination (done on Xario XG, model number SSA 580, Toshiba color Doppler ultrasound scanner) revealed dilated stomach and duodenum till its 3rd part. No calculus was detected in gallbladder or duodenum. Liver and kidneys were normal. There was no ascites. Contrast enhanced computed tomography (CT) scan was performed on a multidetector CT scanner (Model-GE Light speed VCT XTe, whole body 64 Slice CT system, GE Healthcare, Milwaukee, USA). The scan parameters were as follows: 5 mm collimation, 2.5 mm interslice interval, 0.6 seconds rotation time, 15 mm table speed per rotation at 120 KV and automatic mA. Images were acquired in portal venous phase after injecting 90 mL of nonionic iodinated contrast material intravenously at 2.5 mL/s with a pressure injector followed by 20 mL of normal saline flush at the same rate. The multidetector computed tomography (MDCT) scan revealed a cholecysto-duodenal fistula with second part of duodenum. There was a large gallstone measuring 3.2 cm in the proximal 3rd part of the duodenum. Pneumobilia was also seen. Stomach and duodenum were dilated (Figures 1 and 2).

**Figure 3:**
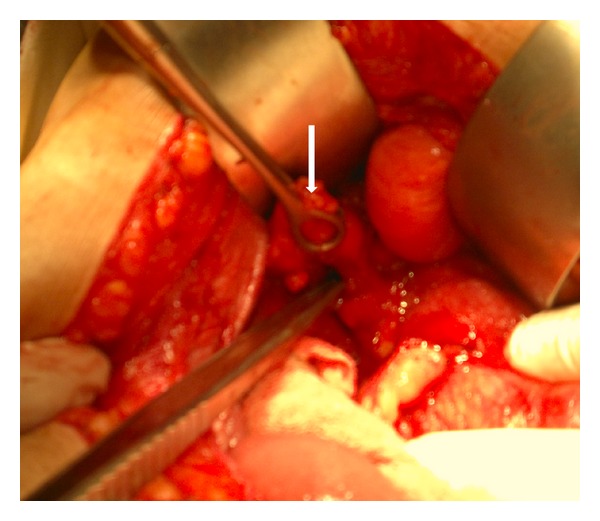
Peroperative image shows the excised fistula (arrow).

**Figure 4:**
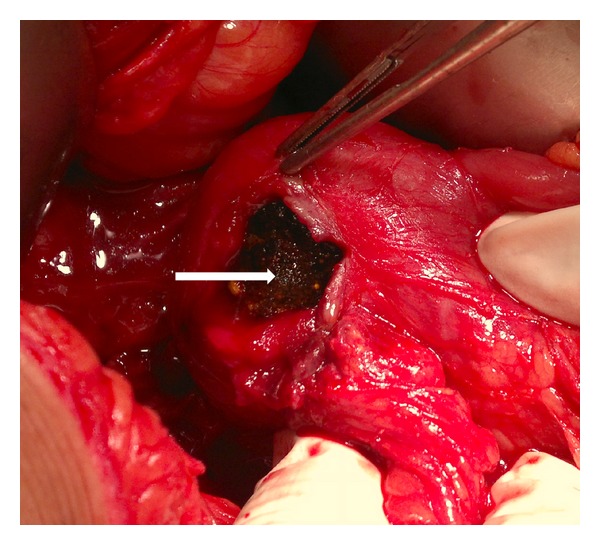
Calculus (arrow) is seen through the duodenotomy incision.

**Figure 5:**
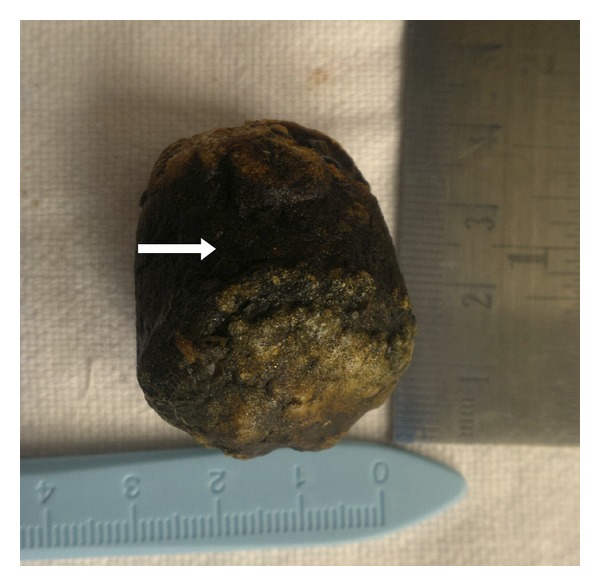
The hardshelled gallstone after surgery.

On laparotomy, multiple adhesions were found between stomach, gallbladder, duodenum, and omentum with inferior surface of liver. Fundus of gallbladder was adherent to 2nd part of duodenum, suggestive of a fistulous tract. The fistulous tract was excised, and the 2nd part of duodenum was opened simultaneously (Figure 3). Duodenotomy was extended, and a black impacted stone was retrieved from the same site measuring 4 × 2.5 cm (Figures 4 and 5). Primary closure of oedematous duodenum was done. Covering gastrojejunostomy and cholecystectomy was also performed simultaneously. Postoperative period was uneventful, and the patient was discharged on 10th postoperative day.

## 3. Discussion

Bouveret's syndrome is gastric outlet obstruction caused by a large gallstone passing into the duodenal bulb through a biliogastric or bilioduodenal fistula [8]. Bouveret's syndrome is reported to occur more frequently in females (65%) with a median age of 69 years. Diagnosis is made usually during the upper GI endoscopy further supported by abdominal ultrasound findings. Computerized tomography is helpful in demonstrating the exact level of obstruction, the biliary site of duodenal fistula, and the status of gallbladder especially in cases of gallbladder rupture [9]. The diagnosis can also be made on plain abdominal radiographs. The classical Rigler's triad of distended stomach, pneumobilia & ectopic radio opaque gallstone is found in approximately one third of cases [10].

 Endoscopic lithotomy is the first-line approach to treatment; however, it may be unsuccessful in some cases particularly with impacted large stones. Smaller stones generally pass through the duodenum and do not cause gastric outlet obstruction [9]. However, larger stones usually get impacted in the duodenum and cause an ischemic ulceration on its wall [7]. Surgical treatment consisting of cholecystectomy and duodenal repair following extraction of the stone through the broken-down cholecysto-duodenal fistula or a separate duodenotomy results in considerable morbidity and mortality [8]. Developments in surgical techniques have reduced the reported mortality rate of 30% before 1968 to 12% in recent years. Accordingly, surgical aim has gradually shifted from a radical procedure, in which the gallbladder is removed and the cholecysto-duodenal fistula is repaired, to a simple approach consisting of enterotomy and stone extraction. Alternatives to surgical lithotomy such as simple endoscopic lithotomy and laser or ESWL have been reported with successful outcomes in some cases [7].

 Enterotomy carries less morbidity rate when compared to duodenotomy particularly in patients with duodenal ulcer due to the erosion of duodenal wall by an impacted large stone. Intraoperative endoscopy may facilitate recognition and removal of remaining stones in the proximal gastrointestinal lumen. Intraoperative ultrasound is advised in cases where the surgeon is unable to localize exactly the site of the remaining stone fragments [2]. In our case, we preoperatively reported the stone as situated in the 3rd part of duodenum causing gastric outlet obstruction, and there was no residual calculus in the gallbladder. The cholecystoduodenal fistula was well demonstrated due to the isotropic reconstruction capability of 64 slice MDCT scanner. This finding changed the operative strategy from a simple enterotomy and stone extraction to a more complicated procedure such as cholecystectomy and duodenal wall repair. In summary, endoscopic lithotripsy and stone extraction should be performed as a first-step treatment in patients with Bouveret's syndrome. When it fails, surgical lithotomy consisting of simple enterotomy may solve the problem. Cholecystectomy and cholecysto-duodenal fistula breakdown is not recommended routinely. However, they can be performed successfully when a definitive preoperative diagnosis is made and the general condition permits the surgeon to undertake such a decision.
